# Influence of GABA_A_ Receptor α Subunit Isoforms on the Benzodiazepine Binding Site

**DOI:** 10.1371/journal.pone.0042101

**Published:** 2012-07-27

**Authors:** Benjamin P. Lüscher, Roland Baur, Maurice Goeldner, Erwin Sigel

**Affiliations:** 1 Institute of Biochemistry and Molecular Medicine, University of Bern, Bern, Switzerland; 2 Laboratoire de Conception et Application de Molécules Bioactives, Unité Mixte de Recherche CNRS, Faculté de Pharmacie, Université de Strasbourg, Illkirch, France; University of Iowa, United States of America

## Abstract

Classical benzodiazepines, such as diazepam, interact with α_x_β_2_γ_2_ GABA_A_ receptors, x = 1, 2, 3, 5 and modulate their function. Modulation of different receptor isoforms probably results in selective behavioural effects as sedation and anxiolysis. Knowledge of differences in the structure of the binding pocket in different receptor isoforms is of interest for the generation of isoform-specific ligands. We studied here the interaction of the covalently reacting diazepam analogue 3-NCS with α_1_S204Cβ_2_γ_2_, α_1_S205Cβ_2_γ_2_ and α_1_T206Cβ_2_γ_2_ and with receptors containing the homologous mutations in α_2_β_2_γ_2_, α_3_β_2_γ_2_, α_5_β_1/2_γ_2_ and α_6_β_2_γ_2_. The interaction was studied using radioactive ligand binding and at the functional level using electrophysiological techniques. Both strategies gave overlapping results. Our data allow conclusions about the relative apposition of α_1_S204Cβ_2_γ_2_, α_1_S205Cβ_2_γ_2_ and α_1_T206Cβ_2_γ_2_ and homologous positions in α_2_, α_3_, α_5_ and α_6_ with C-atom adjacent to the keto-group in diazepam. Together with similar data on the C-atom carrying Cl in diazepam, they indicate that the architecture of the binding site for benzodiazepines differs in each GABA_A_ receptor isoform α_1_β_2_γ_2_, α_2_β_2_γ_2_, α_3_β_2_γ_2_, α_5_β_1/2_γ_2_ and α_6_β_2_γ_2_.

## Introduction

Benzodiazepines are widely used drugs. They exert sedative/hypnotic, anxiolytic, muscle relaxant, and anticonvulsant effects. Benzodiazepines are safe and effective in short term treatments even if some side effects as anterograde amnesia have been reported.

Benzodiazepines act at the major inhibitory neurotransmitter receptor, the γ-aminobutyric acid type A (GABA_A_) receptor. The GABA_A_ receptors are composed of five subunits surrounding a central chloride ion selective channel [1]–[4]. A variety of subunit isoforms of the GABA_A_ receptor has been cloned, leading to a multiplicity of receptor subtypes [1], [2], [5], [6]. The major receptor isoform in mammalian brain consists of α_1_, β_2_, and γ_2_ subunits [6], [7]. Different approaches have indicated a 2α:2β:1γ subunit stoichiometry for this receptor [8]–[13] with a subunit arrangement γβαβα anti-clockwise as seen from the synaptic cleft [11]–[13]. The classical benzodiazepine diazepam binds with high affinity and positively modulates recombinant α_1_β_x_γ_2_ (x = 1, 2, 3), α_2_β_x_γ_2_, α_3_β_x_γ_2_ and α_5_β_x_γ_2_ GABA_A_ receptors. The high affinity binding site has been located between the α and γ subunit and is homologous to the agonist binding site located between the β and α subunit [14], [15]. Even if it is very common in the field to discuss e.g. “α_1_ receptors”, there is good evidence for the fact that many GABA_A_ receptors contain two different α subunit isoforms (e.g. [16]). Exclusively the α subunit adjacent to the γ subunit defines the nature of the benzodiazepine site [17].

It has been demonstrated that the residue H101 within the α_1_ subunit [18] and the homologous residues α_2_H101, α_3_H126 and α_5_H105 are crucial for diazepam potentiation [19]. In α_4_ and α_6_ containing receptors the homologous residue is an arginine rendering these receptors insensitive to classical benzodiazepines [18], [20], [21]. Replacement of arginine by histidine in α_4_ and α_6_ confers benzodiazepine sensitivity [18] and replacement of histidine by arginine in α_1_, α_2_, α_3_ and α_5_ abolishes modulation by diazepam [18], [19].

Pharmacological and behavioral studies of knock-in mice in which the relevant histidine residue has been mutated to an arginine, have led to correlations between the α subunit isoform adjacent to the γ subunit and several of the behavioral effects mediated by benzodiazepines. These studies have revealed that GABA_A_ receptors containing an α_1_ subunit in this position mediate the sedative, the anterograde amnesic and partly the anticonvulsive effects of diazepam [22], [23]. GABA_A_ receptors containing an α_2_ subunit in this position mediate the anxiolytic effect and the myorelaxant effect [24], [25]. GABA_A_ receptors containing either an α_3_ or an α_5_ subunit adjacent to the γ subunit contribute to the myorelaxant actions of benzodiazepines [24], [26], [27].

Diazepam is arranged in the binding pocket of α_1_β_2_γ_2_ receptors such as to allow interaction of a reactive group replacing the –Cl atom with a reactive residue in place of α_1_H101 [28]–[31] and interaction of a reactive group attached to the 3′-atom with a reactive residues in place of α_1_S205 or α_1_S206 [32]. It has also been shown that a reactive residue in place of the –Cl atom interacts with a reactive residue in place the histidine homologous to α_1_H101 in α_2_, α_3_ and the corresponding arginine residue in α_6_ but not to the histidine residue in α_5_ [33].

In addition to the high affinity binding site for benzodiazepines, two low affinity binding sites were identified; one has been described to be located in the lipid bilayer [34] and the second at the α/β subunit interface [33], [35].

**Figure 1:**
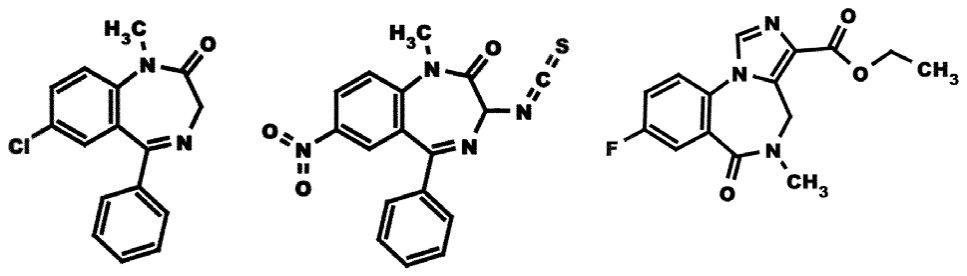
Chemical structures of diazepam, 3-NCS and Ro15–1788.

We used the proximity-accelerated chemical reaction approach to further characterize the architecture of the benzodiazepine binding site in different receptor isoforms. GABA_A_ receptor residues thought to reside in the site were individually mutated to cysteine and combined with a modified benzodiazepine molecule carrying a substituent reactive with cysteine at the 3′ atom (3-NCS, Fig. 1). Direct apposition of target carbon atom of the NCS group and the reactive –S^–^ group of cysteine is expected to lead to a covalent reaction.

**Figure 2:**
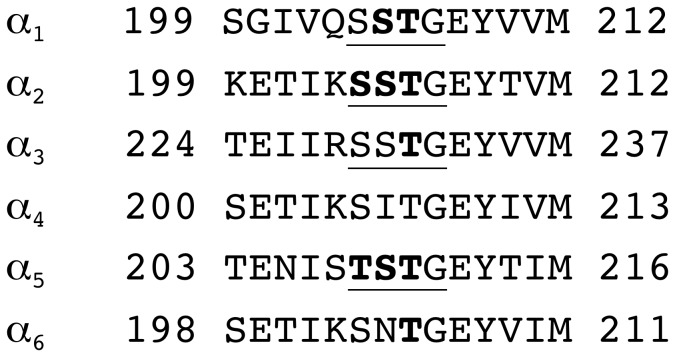
Alignment of Loop C of the α subunits. The underlined residues were individually mutated to cysteine. The bold residues react covalently with 3-NCS. α4 was not investigated and is only shown for comparison.

We studied interaction of 3-NCS compound with amino acid residues 204, 205 and 206 in α_1_β_2_γ_2_ receptors and the homologous residues in α_2_β_2_γ_2_, α_3_β_2_γ_2_, α_5_β_1/2_γ_2_ and α_6_β_2_γ_2_ (Fig. 2), using radioactive ligand binding studies at receptors expressed in HEK-cells and electrophysiological studies, using the two electrode voltage clamp technique at receptors expressed in Xenopus laevis oocytes. In each receptor isoform α_x_β_2_γ_2_ (x = 1, 2, 3, 5, 6), we found a different interaction pattern of the corresponding α subunit with the cysteine reactive compound. This indicates a difference in shape of the benzodiazepine binding site in the region of the 3′ atom of diazepam in different receptor isoforms. We further observed disruption of benzodiazepine binding site in α_1_G207Cβ_2_γ_2_ and α2G207Cβ_2_γ_2_ receptors.

## Methods

### Construction of the Mutated Receptor Subunits

The mutant subunits α_1_S204C, α_1_S205C, α_1_T206C, α_1_G207C, α_2_S204C, α_2_S205C, α_2_T206C, α_2_G207C, α_3_S229C, α_3_S230C, α_3_T231C, α_5_T208C, α_5_S209C, α_5_T210C, α_6_S203C, α_6_N204C and α_6_T205C were prepared using the QuikChange™ mutagenesis kit (Stratagene). For cell transfection, the cDNAs were subcloned into the polylinker of pBC/CMV [36]. This expression vector allows high-level expression of a foreign gene under control of the cytomegalovirus promoter.

### The Cysteine-reactive Compound

The synthesis of the cysteine-reactive compound 3-NCS (Fig. 1) has been described before [32].

### Transfection of GABA_A_ Receptors in HEK293 Cells and Membrane Preparation

cDNAs coding for the α_x_ (x = 1, 2, 3, 6), β_2_, and γ_2_S subunits DNA (20 µg : 20 µg : 20 µg) per 9 cm diameter dish were transfected in human embryonic kidney (HEK) 293 cells (American Type of Culture Collection, MD, USA, CRL 1573) using the calcium phosphate precipitation technique [37]. For α_5_ containing receptors β_2_ was replaced by β_1_. This resulted in higher expression levels. Culturing of cells and membrane preparation were done as described before [30].

### Radioactive Ligand Binding Assay

The properties of the recombinant mutant receptors were only estimated. For this affinity estimate, membranes were re-suspended in phosphate buffer using a Teflon homogenizer. They were incubated in a total volume of 360 µl for 1 h on ice in the presence of [^3^H]Ro15–1788 (78.6 Ci/mmol; PerkinElmer Life Sciences). The final protein concentration was 0.1–1 mg of protein/ml. Total binding was measured at 0.5 and 5 nM [^3^H]Ro15–1788. Nonspecific binding was determined under the same condition but in the presence of 100 µM unlabeled Ro15–1788 and amounted to less than 10% of total binding, except for α_2_S206Cβ_2_γ_2_ and α_5_T208Cβ_2_γ_2_ where it amounted to 10–17%. Expression levels of α_6_β_2_γ_2_ receptors were estimated with [^3^H]Ro15–4513. Membranes were collected by rapid vacuum filtration on GF/C filters. After three washing steps (3 sec each) with 5 ml of phosphate buffer, the filter-retained radioactivity was determined by liquid scintillation counting.

### Detection of a Covalent Reaction

As detailed in previous work [28], [30], [33] this procedure included three steps: incubation of membranes expressing recombinant wild type or mutant receptors with the reactive agent followed by extensive washing of the membranes in order to remove non-reacted compound and a radioactive ligand binding assay to determine residual binding. No covalent reaction would result in 100% residual binding, and 100% covalent reaction would result in 0% residual binding.

Briefly, the membranes were re-suspended in phosphate buffer (100 mM KCl, 10 mM KH_2_PO4, 0.1 mM EDTA, pH 7.4) using a Glass/Teflon homogenizer. 0.1–1.0 mg/mL of protein were incubated in a total volume of 360 µL with either 10 µM (determination of degree of covalent reaction) or several concentrations of 3-NCS for 30 min on ice. Membranes were collected with rapid filtration on a round 7 mm diameter glass fiber filter (GF/C; Whatman) that was placed on a round 24 mm diameter glass fiber filter (GF/C; Whatman), both pre-washed with phosphate buffer. The reaction of 3-NCS with the receptor was stopped by washing of the filters six times with 5 mL phosphate buffer, each. The small filters with the deposited membranes were incubated in 0.12 mL phosphate buffer containing 5 nM [^3^H]Ro15–1788. After 30 min the 7 mm filter was placed on a 24 mm filter and washed six times with 5 mL phosphate buffer each. Radioactivity was determined by liquid scintillation counting. Non-specific binding was determined in the presence of 100 µM Ro 15–1788. In control experiments, washing efficiency was estimated by placing radioactivity on the small filter. More than 99.95% of the radioactivity was removed (not shown).

Concentration response curves for 3-NCS were fitted with the equation C(c)  =  C_max_/(1+(EC_50_/c)), where c is the concentration of 3-NCS, EC_50_ the concentration of 3-NCS where half maximal covalent reaction was observed, C_max_ is the maximal extent of the covalent reaction and C the measured extent of the covalent reaction.

### Expression of GABA_A_ Receptors in Xenopus Oocytes

Capped cRNAs were synthesized (Ambion, Austin, TX, USA) from the linearized plasmids with a cytomegalovirus promotor (pCMVvectors) containing the different subunits, respectively. A poly-A tail of about 400 residues was added to each transcript using yeast poly- A polymerase (United States Biologicals, Cleveland, OH, USA). The concentration of the cRNA was quantified on a formaldehyde gel using Radiant Red stain (Bio-Rad) for visualization of the RNA. Known concentrations of RNA ladder (Invitrogen) were loaded as standard on the same gel. cRNAs were precipitated in ethanol/isoamylalcohol 19∶ 1, the dried pellet dissolved in water and stored at −80°C. cRNA mixtures were prepared from these stock solutions and stored at −80°C. Xenopus laevis oocytes were prepared, injected and defolliculated as described previously [38], [39]. They were injected with 50 nL of the cRNA solution containing wild type or mutated α_1_ or α_2_, α_3_, α_5_, α_6_ and wild type β_2_ and γ_2_ subunits at a concentration of 10 nM : 10 nM : 50 nM [40] and then incubated in modified Barth’s solution at +18°C for at least 24 h before the measurements.

### Functional Characterization of the GABA_A_ Receptors

Currents were measured using a modified two-electrode voltage clamp amplifier Oocyte clamp OC-725 (Warner Instruments) in combination with a XY-recorder (90% response time 0.1s) or digitized at 100 Hz using a PowerLab 2/20 (AD Instruments) using the computer programs Chart (ADInstruments GmbH, Spechbach, Germany). Tests with a model oocyte were performed to ensure linearity in the larger current range. The response was linear up to 15 µA.

Electrophysiological experiments were performed by using the two-electrode voltage clamp method at a holding potential of −80 mV. The perfusion medium contained 90 mM NaCl, 1 mM KCl, 1 mM MgCl_2_, 1 mM CaCl_2_, and 5 mM Na-HEPES (pH 7.4) and was applied by gravity flow 6 ml/min. The perfusion medium was applied through a glass capillary with an inner diameter of 1.35 mm, the mouth of which was placed about 0.4 mm from the surface of the oocyte. Allosteric modulation via the benzodiazepine site was measured at a GABA concentration eliciting 2–5% of the maximal GABA current amplitude. GABA was applied for 20 s alone or in combination with allosteric compound. 6 ml of the covalent reacting compound was applied to the oocyte and incubated for 3 min while stopping the flow of the perfusion medium. Modulation of GABA currents was expressed as (I_(modulator + GABA)_/I_GABA_ –1) * 100%. The perfusion system was cleaned between drug applications by washing with DMSO to avoid contamination.

## Results

We wanted to derive information on part of the benzodiazepine binding pockets in different GABA_A_ receptor isoforms. For this purpose, we characterized the covalent interaction of a benzodiazepine-like compound with α_x_β_1/2_γ_2_ (x = 1, 2, 3, 5, 6) receptors containing a cysteine mutation in residues α_1_S204C, α_1_S205C and α_1_T206C and homologous positions in α_2_, α_3_, α_5_ and α_6_. The β_2_ subunit was used throughout except for expression of α_5_ containing receptors in HEK-cells where β_1_ was used instead to achieve higher expression levels. α_1_G207C and the homologous mutation α_2_G207C were also prepared. These mutated subunits did not confer to the expressed receptors high affinity [^3^H]Ro 15–1788 or [^3^H]flunitrazepam binding nor functional modulation by diazepam (not shown). This indicates that mutation in this position disrupts the binding site for benzodiazepines.

### The Cysteine Reactive Compound

We used a modified nitrazepam molecule carrying a cysteine reactive isothiocyanate substituent at the C-3 carbon (3-NCS; Fig. 1). 3-NCS was able to displace [^3^H]Ro15–1788 or [^3^H]flunitrazepam from wild-type α_1_β_2_γ_2_ receptors. The K_i_ values were 340±16 nM and 240±55 nM [32], respectively. The K_i_ values for displacement of [^3^H]Ro 15–1788 by 3-NCS in α_2_β_2_γ_2_ were 1550±250 nM (n = 3) and in α_5_β_1_γ_2_ receptors 9840±1480 nM (n = 3). The determination of K_i_ values was based on the K_d_ values of 2.1±0.5 nM and 1.5±1.2 nM, respectively. The covalent reaction of 3-NCS with α_1_mβ_2_γ_2_ (m = S205C, T206C) receptor at the binding and functional level has previously been described [32].

### Binding Properties of the GABA_A_ Receptor Carrying a Cysteine Point Mutation

The homologous residues to α_1_S204, α_1_S205 and α_1_T206 in α_2_, α_3_, α_5_ and α_6_ were mutated individually to cysteine. α_1_ and α_2_ were expressed in combination with β_2_ and γ_2_ subunits, α_5_ for reasons mentioned above together with β_1_ and γ_2_ subunits. All these mutated α_m_β_2_γ_2_ receptors bound [^3^H]Ro 15–1788 with an estimated affinity between 0.08 and 7.5 nM (data not shown). No specific binding was detected in α_ 3_ containing receptors (data not shown). α_6_T205Cβ_2_γ_2_ bound [^3^H]Ro15–4513 with an estimated affinity 4 nM.

### Covalent Reaction of 3-NCS at the Binding Level

First we tested reactivity of 3-NCS using a radioactive ligand binding assay. 3-NCS is expected to first occupy its binding site reversibly. Upon proper apposition of the –SH group of the cysteine from the mutated receptors with the –C atom of the NCS group from the 3-NCS compound, this is followed by covalent reaction. This reaction was determined at 10 µM concentration of 3-NCS. Preliminary experiments showed that at this concentration and at 1 µM 3-NCS, covalent reaction was reaching a maximum within 15 sec (not shown). Covalent reaction did not reach completion. This observation was made before and has been interpreted to indicate covalent reaction at a low affinity benzodiazepine binding site at the α/β subunit interface, which prevents covalent reaction on the classical benzodiazepine binding site [33]. Alternatively or in addition, the reactive compound may be consumed in non-specific reactions. Non-covalently reacted compound was removed by filtration (see methods). A covalent reaction of 3-NCS with a mutated cysteine residue is expected to prevent reversible binding of the [^3^H]Ro15–1788. If no covalent reaction occurs, the binding site should still be available for reversible binding. α_5_ containing receptors were expressed together with β_1_ instead of β_2_. α_3_ containing receptors did not express in HEK293-cells. As α_6_ subunit containing receptors do not bind [^3^H]Ro15–1788 with high affinity, we used [^3^H]Ro15–4513 instead.

**Figure 3:**
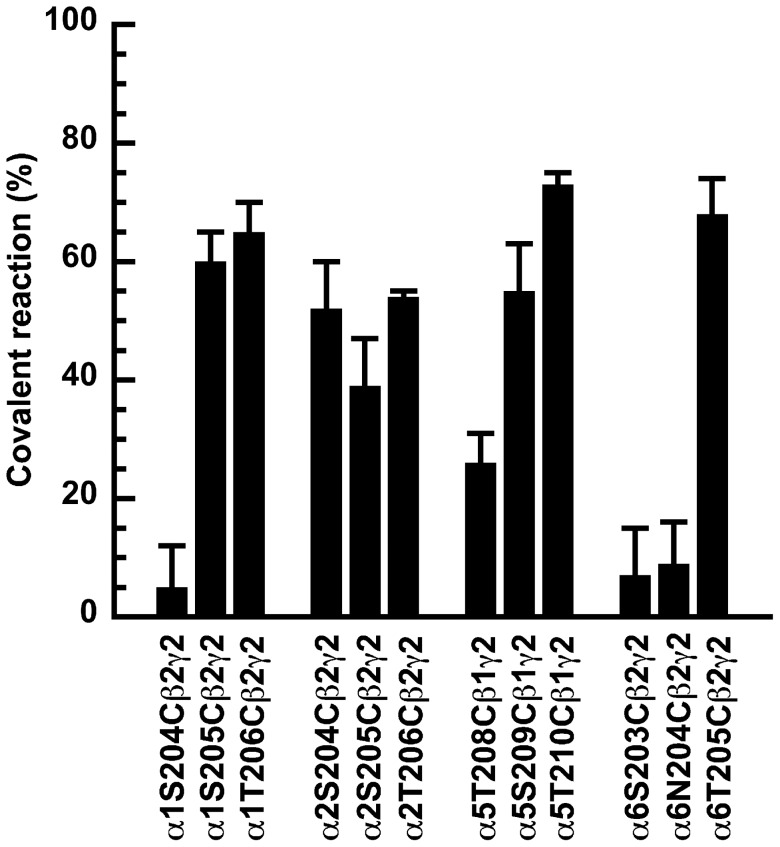
Covalent reaction at the binding level. Extent of covalent reaction of 3-NCS at α_1_S204Cβ_2_γ_2_, α_1_S205Cβ_2_γ_2_, α_1_T206Cβ_2_γ_2_ mutant GABA_A_ receptors and homologous receptors formed with α_2_, α_3_, and α_6_. α_5_ was expressed together with β_1_ and γ_2_ subunits for reasons mentioned in the results section. Receptors were expressed in HEK-293 cells, membranes harvested and exposed to 10 µM 3-NCS. After incubation, the residual 3-NCS was removed by filtration. [^3^H]Ro15–1788 was used as radioactive ligand to determine the residual binding. Covalent binding is expressed as 100% minus % residual binding. Data are shown as mean ±SD for three experiments each (triplicates of each point in each experiment).

Figure 3 compares the percentage of covalent reaction, in mutated α_1_, α_2_, α_5_ and α_6_ containing receptors. For the α_1_β_2_γ_2_ receptor isoform cysteine mutation in residue S205 and T206 resulted in covalent reaction, while S204C did not covalently react with 3-NCS [32]. For α_2_β_2_γ_2_ receptor isoform the cysteine mutated residues homologous to α_1_S204, α_1_S205 and α_1_T206 showed covalent reaction amounting to 52%±8% (n = 3), 39%±8% (n = 3) and 54%±1% (n = 3). For α_5_β_1_γ_2_ receptors the cysteine mutated receptors homologous to α_1_S204, α_1_S205 and α_1_T206 reacted covalently amounting to 26%±5% (n = 3), 54%±8% (n = 3) and 73%±2% (n = 3). In the α_6_β_2_γ_2_ receptor isoform only the cysteine mutation homologous to α_1_T206 reacted covalently with 3-NCS amounting to 68% ±6% (n = 3).

### Concentration Dependence of the Covalent Reaction

**Figure 4:**
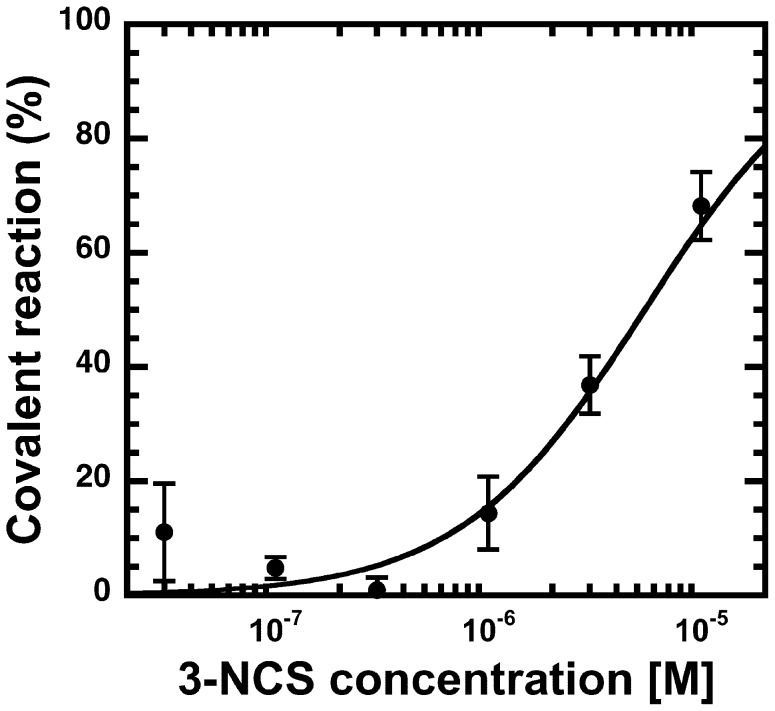
Concentration dependence at the binding level. Concentration dependence of the covalent reaction of the 3-NCS at α_6_T205Cβ_2_γ_2_ mutant GABA_A_ receptors. Extent of covalent reaction was determined at different concentrations of 3-NCS as indicated below Fig. 3. Data are shown as mean ±SD for three experiments each (triplicates of each point in each experiment).

We next investigated the concentration dependence of the covalent reaction. Mutated receptors were exposed for 30 min to different concentrations of 3-NCS. Figure 4 documents such a concentration dependence in α_6_T205Cβ_2_γ_2_ receptors. The concentration dependence was also determined in mutated α_1_S204Cβ_2_γ_2_, α_1_S205Cβ_2_γ_2_, α_1_T206Cβ_2_γ_2_ and homologous mutations in α_2_, α_5_ and α_6_ containing receptors. Table 1 documents the calculated EC_50_ values of the covalent reaction, which are between 0.08 µM and 6.6 µM. For α_1_S204Cβ_2_γ_2_, α_6_S203Cβ_2_γ_2_, α_6_N204Cβ_2_γ_2_ the EC_50_ values could not be determined because we could not detect covalent reaction with 3-NCS.

**Table 1:** Concentration dependence of the covalent reaction at mutant receptors at the binding level.

mutated receptor	mutation homologous to	EC_50_ (µM)
α_1_S204Cβ_2_γ_2_		n.r.
α_1_S205Cβ_2_γ_2_		0.63±0.18
α_1_T206Cβ_2_γ_2_		0.082±0.028
α_2_S204Cβ_2_γ_2_	α_1_S204C	3.2±2.3
α_2_S205Cβ_2_γ_2_	α_1_S205C	6.6±5.4
α_2_T206Cβ_2_γ_2_	α_1_S206C	0.19±0.056
α_5_T208Cβ_1_γ_2_	α_1_S204C	0.39±0.21
α_5_S209Cβ_1_γ_2_	α_1_S205C	4.1±2.2
α_5_T210Cβ_1_γ_2_	α_1_S206C	0.45±0.15
α_6_S203Cβ_2_γ_2_	α_1_S204C	n.r. a
α_6_N204Cβ_2_γ_2_	α_1_S205C	n.r. a
α_6_T205Cβ_2_γ_2_	α_1_S206C	5.4±0.80^a^

Left column: mutated receptors. Middle column: homology of the mutations to α_1_. Right column EC_50_ is given as mean ±SD for three to four experiments where each point in the dose-response curve was determined in triplicates. The receptors were exposed to increasing concentrations of 3-NCS compound for 30 min on ice and extensively washed. Residual binding was determined using [^3^H]Ro15–1788 as radioactive ligand and converted to percentage of binding sites covalently reacted. All wild type receptors showed no covalent reaction. n.r.: no covalent reaction.

a [^3^H]15–4513 was used as a radioactive ligand.

Residue 206 is unique in that all the investigated subunit isoforms do show covalent reaction. In each case the absolute level of the reaction is relatively high (Fig. 3). It should be noted that residue 206 in the α_1_ subunit and homologous residues in α_2_ and α_5_ show a very high apparent affinity in the reaction with 3-NCS while the corresponding residue in α_6_ shows a lower apparent affinity (F-test; p<0.01). In α_1_, α_2_ and α_5_, the residue identical to or homologous to residue 206 in the α_1_ subunit has a lower EC_50_ than residue 205 in the corresponding α subunit (F-test; p<0.01). Residue 205 in the α_1_ subunit shows a higher apparent affinity in the reaction with 3-NCS than the homologous residue in α_2_ (F-test; p<0.02).

### Irreversible Reaction of 3-NCS at the Functional Level

**Figure 5:**
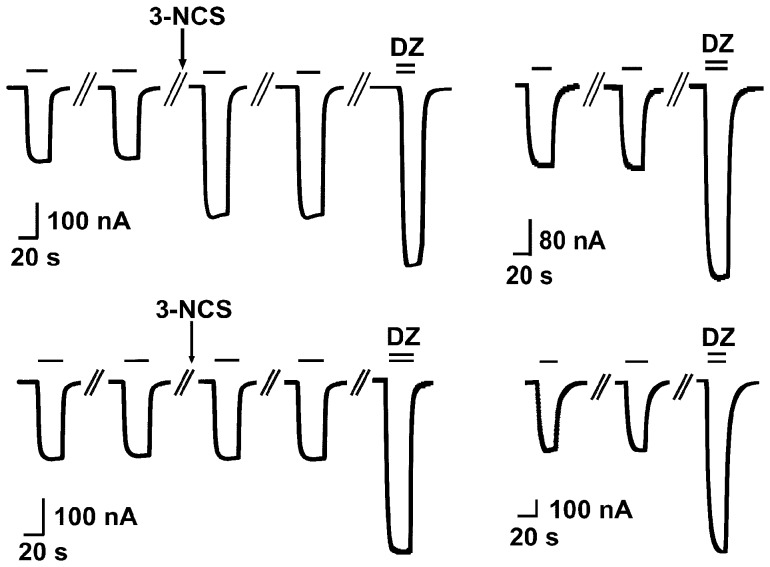
Covalent reaction at the functional level. Exposure of mutated α_1_S205Cβ_2_γ_2_ receptors to 3-NCS results in a covalent reaction. a) Receptors were exposed twice to 0.5 µM GABA followed by a washing period and a 3 min incubation in 10 µM 3-NCS. After this treatment GABA was applied twice with a 3 min interval. In some experiments GABA was applied up to six times. Thus, the current elicited by GABA was stimulated irreversibly. Application of 0.5 µM GABA in combination with 1 µM diazepam results in a further stimulation of the partially irreversibly reacted receptors that was smaller than that seen upon b) direct exposure to diazepam in independent experiments. Exposure of α_1_T204Cβ_2_γ_2_ receptors 3-NCS does not result in a covalent reaction. c) Receptors were exposed twice to 0.5 µM GABA followed by a 3 min incubation in 10 µM 3-NCS. Application of 0.5 µM GABA in combination with 1 µM diazepam results in stimulation similar to the stimulation seen in an oocyte that had not been previously exposed to 3-NCS (Fig. 4d).

We also studied the covalent reaction at the functional level. All wild type receptors failed to show any changes in the current amplitude elicited by GABA, while some mutant receptors did. An example of this experiment is shown in Figure 5a for the α_1_S205Cβ_2_γ_2_ mutant receptor. 3-NCS led to an irreversible increase in the current amplitude and the relative stimulation by diazepam was smaller than observed in naïve oocytes (Fig. 5b). We interpret this as evidence for a covalent reaction. Fig. 5c shows the same experiment at the mutant receptor α_1_T204Cβ_2_γ_2_. 3-NCS did not increase the current amplitude and diazepam stimulated to the same extent as in naïve oocytes (Fig. 5d). Clearly, this mutant receptor did not show any covalent reaction. As α_6_ containing receptors did not respond to diazepam, we used 1 µM abercarnil instead.

Exposure of mutant receptors α_1_S205Cβ_2_γ_2_, α_1_T206Cβ_2_γ_2_, α_2_T205Cβ_2_γ_2_ (mutation homologous to α_1_S205), α_2_T206Cβ_2_γ_2_ (α_1_T206), α_3_T231Cβ_2_γ_2_ (α_1_T206), α_5_T208Cβ_2_γ_2_ (α_1_S204), α_5_T209Cβ_2_γ_2_ (α_1_S205), and possibly α_3_S230Cβ_2_γ_2_ (α_1_S205) and α_6_T205Cβ_2_γ_2_ (α_1_T206), to 3-NCS resulted in an irreversible positive allosteric modulation (Table 2) indicating a covalent reaction, the adduct acting as a positive allosteric modulator. If only a fraction of the receptors react with 3-NCS, diazepam is expected to stimulate the remaining receptors. If 3-NCS has a similar allosteric effect as diazepam the combined stimulations of 3-NCS and diazepam should be the same as stimulation by diazepam alone. This is the case for most mutated receptors studied. Exceptions are α_1_T206Cβ_2_γ_2_, α_3_T231Cβ_2_γ_2_ (homologous to α_1_T206), α_5_T208Cβ_2_γ_2_ (α_1_S204) and α_5_T210Cβ_2_γ_2_ (α_1_T206). In the last two cases, results may be explained if 3-NCS acts at these mutated receptors as a partial modulator and an antagonist, respectively, at the benzodiazepine site. In the first two cases combined stimulation by 3-NCS and diazepam is larger than expected from diazepam alone. The latter seems in both cases reduced by the mutation for unknown reasons. We can only hypothesize that covalent reaction with 3-NCS at the α/β subunit interface may allosterically stimulate the reaction to diazepam. It is interesting to note that at the functional level the identical residues were identified to react covalently as at the binding level. This in spite of the use of Ro15–1788 to detect covalent reaction at the binding level and the use of diazepam at the functional level. The only exception is α_2_S204C. We have no explanation for this discrepancy. Differences in the experimental conditions used in binding and functional experiments (total lipid content, lipid composition and temperature) may be responsible.

**Table 2:** Covalent reaction at the functional level.

Mutated receptor	Mutation homolo-gous to	DZ	n	3-NCS	p	3-NCS +DZ	n
α_1_S204Cβ_2_γ_2_^+^		126±9	3	4±4	n.s.	115±34	3
α_1_S205Cβ_2_γ_2_		113±7	3	60±43	<0.03	131±32	3
α_1_T206Cβ_2_γ_2_		82±18	6	119±51	<0.01	204±62	4
α_2_S204Cβ_2_γ_2_^+^	α_1_S204C	215±42	3	1±10	n.s.	218±30	3
α_2_S205Cβ_2_γ_2_	α_1_S205C	191±53	3	35±10	<0.01	177±25	3
α_2_T206Cβ_2_γ_2_	α_1_S206C	148±8	3	53±29	<0.01	168±54	3
α_3_S229Cβ_2_γ_2_	α_1_S204C	l.e.		l.e.		l.e.	
α_3_S230Cβ_2_γ_2_	α_1_S205C	149±44	3	15±11	n.s.	158±44	3
α_3_T231Cβ_2_γ_2_	α_1_S206C	88±45	3	28±16	<0.03	165±8	3
α_5_T208Cβ_2_γ_2_	α_1_S204C	129±38	7	23±18	<0.05	83±25	5
α_5_S209Cβ_2_γ_2_	α_1_S205C	103±3	3	46±17	<0.01	140±20	3
α_5_T210Cβ_2_γ_2_	α_1_S206C	82±14	3	10±5	n.s.	47±8	3
α_6_S203Cβ_2_γ_2_^+^	α_1_S204C	50±20*	3	0±2	n.s.	57±21*	3
α_6_N204Cβ_2_γ_2_^+^	α_1_S205C	70±40*	3	−4±6	n.s.	71±40*	3
α_6_T205Cβ_2_γ_2_	α_1_S206C	48±6*	3	14±8	<0.05	58±21*	3

Mutated GABA_A_ receptors were expressed in Xenopus oocytes. Allosteric stimulation by 1 µM diazepam was determined (column labelled DZ (diazepam)) at EC_2–5_ for GABA. Data are given as % allosteric modulation. In independent experiments oocytes were exposed to GABA followed by 10 µM 3-NCS and after removing non-covalently reacted 3-NCS, allosteric stimulation was determined (column labelled 3-NCS). Subsequently the same oocyte was exposed to 1 µM diazepam (column labelled 3-NCS + diazepam) and allosteric stimulation was determined as compared to the initial application of GABA. *1µM Abecarnil was used. l.e. low expression, expressed currents were too small for measurement of covalent effects. p was determined with the one-way ANOVA followed by a post-hoc Dunnett’s test where the non-responsive receptors indicated with (^+^) served as one of the samples (mean ± mean SD, 0.25±5.5, n = 4). Data are given as mean ±SD.

## Discussion

The most common GABA_A_ receptors contain two identical or different α subunits [6]. Receptors containing different α subunit isoforms adjacent to the γ subunit mediate different effects of classical benzodiazepines. α_1_, α_2_, α_3_ or α_5_ are required in this position for the action of classical benzodiazepines. In order to be able to rationally design receptor subtype specific drugs more knowledge on the difference of the interaction of different receptor subtypes with classical benzodiazepines is required.

**Figure 6:**
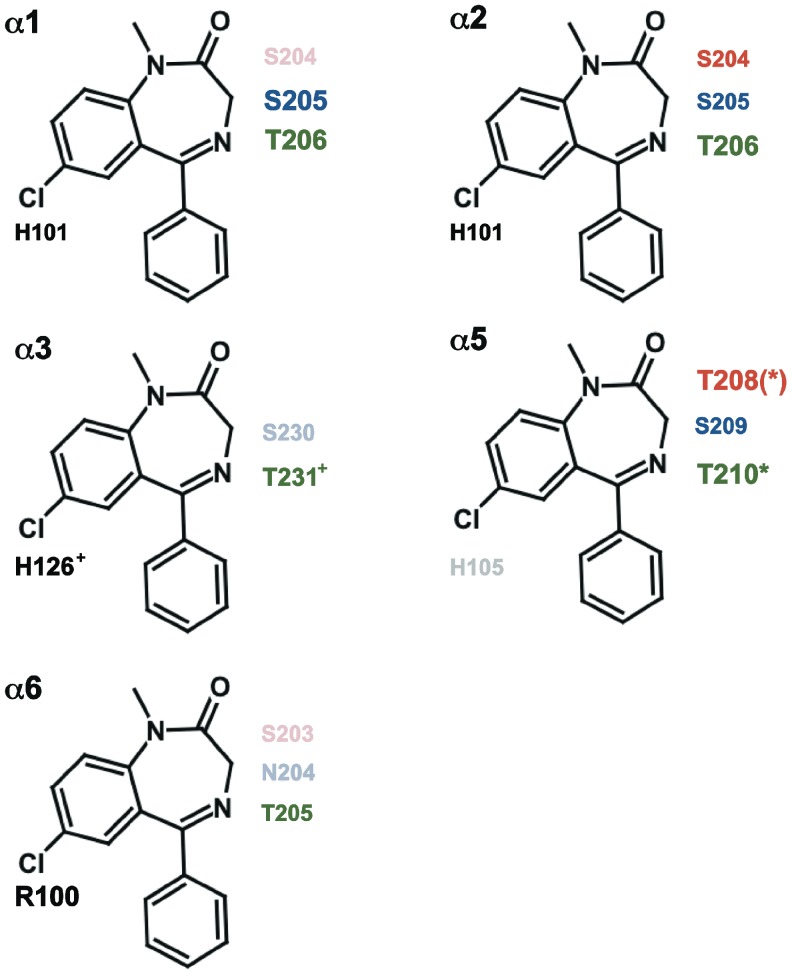
Summary. The results obtained with NCS [33] and the results obtained with 3-NCS described here are summarized. For each receptor isoform α_x_β_1/2_γ_2_ (x = 1, 2, 3, 5, 6) amino acid residues homologous to 101 in α_1_ are shown in black, those homologous to 204 are shown in red, those homologous to 205 are shown in blue and those homologous to 206 are shown in green. Residue 101 is located near to the C atom carrying the Cl atom in diazepam, and 204, 205 and 206 are near to C atom adjacent to the keto group. The residues showing covalent reaction are shown in full color and those showing no reaction coloured in reduced saturation. The residues in larger font size react with a high apparent affinity. The residues marked with * and (*) indicate an antagonistic effect and a partial modulatory effect by 3-NCS on the corresponding α_5m_β_2_γ_2_ receptors, respectively. The residues marked with ^+^ indicate that the corresponding α_3m_β_2_γ_2_ receptors were only investigated at the functional level.

We aimed at finding differences or similarities in the shape of the benzodiazepine binding site in different receptor isoforms. We used the proximity-accelerated chemical coupling reaction at the binding and functional level. A Cysteine-mutated receptor is combined with a chemical modified cysteine reactive binding site ligand. Indentification of a covalent reaction implies apposition of the –S atom of cysteine and the reactive –C atom of the ligand [41]. After previous work in the α_1_H101 region [33], where we found that a NCS group introduced into diazepam at the –Cl position interacts subtly different with α_1_, α_2_, α_3_ and α_6_, but only very weakly with α_5_, we concentrated here on the C atom adjacent to the keto group in diazepam. We have previously shown that α_1_S205Cβ_2_γ_2_ and α_1_T206Cβ_2_γ_2_ receptors but not α_1_S204Cβ_2_γ_2_ receptors interact covalently with 3-NCS [32]. Now homologous mutations were introduced into other α subunit isoforms.

We studied the covalent reaction at the level of radioactive ligand binding at receptors expressed in transfected HEK 293 cells and at the functional level characterizing chloride current mediated by receptors expressed in Xenopus oocytes. In general, we found very good agreement of the results obtained by the two strategies. In case covalent reaction occurs not in the binding pocket, but on the access pathway of 3-NCS, we would not expect an allosteric modulation of the corresponding receptor. Thus the agreement between the strategies can be taken as evidence for a reaction in the binding pocket.

In the following we discuss in sequence our observations for the position homologous to α_1_S204, to α_1_S205, to α_1_T206 and to α_1_G207 in α_x_β_2_γ_2_ (x = 1, 2, 3, 5, 6) receptors.

Position homologous to α_1_S204: At the binding level, mutated α_2_β_2_γ_2_ and α_5_β_1_γ_2_ showed covalent reaction, while α_1_β_2_γ_2_ and α_6_β_2_γ_2_ did not. At the functional level, exclusively mutated α_5_β_2_γ_2_ showed covalent reaction. We have no explanation for the discrepancy concerning α_2_β_2_γ_2_. Possibly, 3-NCS acts as an antagonist here and covalent reaction at the α/β subunit interface promotes stimulation by diazepam. α_3_β_2_γ_2_ could not be expressed at sufficient extent to determine reaction levels.

Position homologous to α_1_S205: At the binding level, mutated α_1_β_2_γ_2_, α_2_β_2_γ_2_ and α_5_β_1_γ_2_ showed covalent reaction, while mutated α_6_β_2_γ_2_ did not. At the functional level, the same result was obtained. In addition, α_3_β_2_γ_2_ might have reacted covalently at the functional level, but reaction was at the threshold for significance.

Position homologous to α_1_T206: At the binding level, all mutated receptors investigated α_1_β_2_γ_2_, α_2_β_2_γ_2_, α_5_β_1_γ_2_ and α_6_β_2_γ_2_ showed covalent reaction. At the functional level, the same result was obtained except for possibly α_6_β_2_γ_2_, which is at the threshold for significance. In addition, mutated α_3_β_2_γ_2_ reacted covalently.

Position homologous to α_1_G207: At the binding and at the functional level mutated α_1_β_2_γ_2_ and α_2_β_2_γ_2_ receptors were missing the benzodiazepine binding site. In functional experiments currents induced by GABA were not affected by the mutations. All other receptor isoforms were not tested.

In summary there is evidence for covalent reaction of the α_1_ subunit in residues **205** and **206**; of the α_2_ subunit in residues 204, 205 and **206**; of the α_3_ subunit at least in residue 206 and possibly 205; of the α_5_ subunit in residues **204**, 205 and **206**; and of the α_6_ subunit exclusively in residue 206. The residues highlighted in bold face react with a high apparent affinity. It is interesting to note that the residue homologous to 206 reacts covalently in all receptors. Unexpectedly, the α_2_ subunit showed a similar reactivity pattern as the α_5_ subunit.

These observations should be combined with data on another region of the benzodiazepine binding pocket. The region of the –Cl atom in diazepam has previously been investigated in different GABA_A_ receptor isoforms [33]. A molecule made reactive in this position interacted best with α_6_ containing receptors and very little with α_5_ containing receptors while the variants with α_1_, α_2_, and α_3_ were intermediate.

The combined set of data is visualized in Fig. 6. The size of the lettering of the residues correlates qualitatively with the affinity of the covalent reaction. Our observations will help in modelling of the benzodiazepine binding pocket in different α subunit containing isoforms of the GABA_A_ receptor.
